# Biodistribution and imaging of an hsp90 ligand labelled with ^111^In and ^67^Ga for imaging of cell death

**DOI:** 10.1186/s13550-020-0590-x

**Published:** 2020-01-20

**Authors:** Ivan Ho Shon, Divesh Kumar, Chithradevi Sathiakumar, Paula Berghofer, Khang Van, Andrew Chicco, Philip J. Hogg

**Affiliations:** 1 Department of Nuclear Medicine and PET, Prince of Wales Hospital, Randwick, 2031 NSW Australia; 2 The Centenary Institute, NHMRC Clinical Trials Centre, Sydney Medical School, University of Sydney, Sydney, NSW 2006 Australia; 3 Prince of Wales Clinical School, University of New South Wales, Sydney, 2052 NSW Australia; 4 Department of Nuclear Medicine and PET, Fiona Stanley Hospital, Murdoch, 6150 WA Australia; 5 Department of Nuclear Medicine and PET, Liverpool Hospital, Liverpool, NSW 2170 Australia; 6 LifeSciences Division, Australian Nuclear Science and Technology Organisation, New Illawarra Road, Lucas Heights, Sydney, NSW 2234 Australia; 7 Department of Medical Physics, Westmead Hospital, Westmead, NSW 2145 Australia

**Keywords:** Cell death, Indium-111, Gallium-67, Radionuclide imaging

## Abstract

**Background:**

4-(N-(S-glutathionylacetyl)amino) phenylarsonous acid (GSAO) when conjugated at the γ-glutamyl residue with fluorophores and radio-isotopes is able to image dead and dying cells in vitro and in vivo by binding to intracellular 90-kDa heat shock proteins (hsp90) when cell membrane integrity is compromised. The ability to image cell death has potential clinical impact especially for early treatment response assessment in oncology. This work aims to assess the biodistribution and tumour uptake of diethylene triamine pentaacetic acid GSAO labelled with ^111^In ([^111^In]In-DTPA-GSAO) and 1,4,7,10-tetraazacyclododecane-1,4,7,10-tetraacetic acid GSAO labelled with ^67^Ga ([^67^Ga]Ga-DOTA-GSAO) in a murine subcutaneous tumour xenograft model and estimate dosimetry of [^67^Ga]Ga-DOTA-GSAO.

**Results:**

There was good tumour uptake of both [^111^In]In-DTPA-GSAO and [^67^Ga]Ga-DOTA-GSAO (2.44 ± 0.26% injected activity per gramme of tissue (%IA/g) and 2.75 ± 0.34 %IA/g, respectively) in Balb c nu/nu mice bearing subcutaneous tumour xenografts of a human metastatic prostate cancer cell line (PC3M-luc-c6). Peak tumour uptake occurred at 2.7 h post injection. [^111^In]In-DTPA-GSAO and [^67^Ga]Ga-DOTA-GSAO demonstrated increased uptake in the liver (4.40 ± 0.86 %IA/g and 1.72 ± 0.27 %IA/g, respectively), kidneys (16.54 ± 3.86 %IA/g and 8.16 ± 1.33 %IA/g) and spleen (6.44 ± 1.24 %IA/g and 1.85 ± 0.44 %IA/g); however, uptake in these organs was significantly lower with [^67^Ga]Ga-DOTA-GSAO (*p* = 0.006, *p* = 0.017 and *p* = 0.003, respectively). Uptake of [^67^Ga]Ga-DOTA-GSAO into tumour was higher than all organs except the kidneys. There was negligible uptake in the other organs. Excretion of [^67^Ga]Ga-DOTA-GSAO was more rapid than [^111^In]In-DTPA-GSAO. Estimated effective dose of [^67^Ga]Ga-DOTA-GSAO for an adult male human was 1.54 × 10^− 2^ mSv/MBq.

**Conclusions:**

[^67^Ga]Ga-DOTA-GSAO demonstrates higher specific uptake in dead and dying cells within tumours and lower uptake in normal organs than [^111^In]In-DTPA-GSAO. [^67^Ga]Ga-DOTA-GSAO may be potentially useful for imaging cell death in vivo. Dosimetry estimates for [^67^Ga]Ga-DOTA-GSAO are acceptable for future human studies. This work also prepares for development of ^68^Ga GSAO radiopharmaceuticals.

## Background

Cell death is a fundamental process in health and many disease states. In oncology, dysregulation of cell death by apoptosis is recognized as a “hallmark of cancer” [1] and many cancer therapies, especially cytotoxic chemotherapy and radiotherapy, induce cell death by apoptosis [2, 3]. The ability to image cell death in vivo represents a potentially useful technique for the assessment of many diseases and, in particular, would potentially allow accurate prediction of treatment response in oncology, much earlier than existing imaging technologies.

There have been several approaches to imaging cell death. During cell death, a key event is the externalization of the phospholipid, phosphatidylserine (PS), to the outer leaflet of the cell membrane [4]. Annexin V is a protein that exhibits calcium-dependent binding to PS. The first attempts to directly image cell death were with Annexin V labelled with ^99m^Tc and also ^18^F. [^99m^Tc]Tc-Annexin V uptake has been shown to be correlated with survival following treatment [5, 6] and also predicts cytological apoptosis [7]. However, other studies have failed to demonstrate a relationship between [^99m^Tc]Tc-Annexin V uptake and apoptosis [8]. Annexin V-based radiopharmaceuticals demonstrate high levels of uptake in normal tissues (especially kidneys and liver) which may limit the ability of these agents to assess apoptosis in these organs [9]. Other approaches to imaging PS externalization during cell death have included radiolabelled synaptotagmin I and small molecule binders of PS; however despite this, [^99m^Tc]Tc-Annexin V still remains the tracer of choice for imaging cell death via PS targeting [10].

A second approach to imaging cell death has been the targeting of activated caspase 3. Radiolabelled peptides and small molecules have been investigated for imaging of activated caspase 3/7 during cell death [11]. One of these, [^18^F]ICMT-11, has been assessed in human trials. A first in human biodistribution study of [^18^F]ICMT-11 demonstrated high levels of activity in the hepatobiliary system and small and large intestine due to excretion [12], which may preclude assessment of cell death in the abdomen and pelvis. In a follow-up study in patients with breast and lung cancer, no increase in activated caspase 3 following chemotherapy was observed, which was attributed to the relatively small increase in activated caspase 3/7 in response to chemotherapy [13]. Other approaches have included [^18^F]ML-10- [14] and ^111^In-labelled monoclonal antibodies to the La antigen [15].

4-(N-(S-glutathionylacetyl)amino) phenylarsonous acid (GSAO) is a peptide trivalent arsenical that activates the mitochondrial permeability transition pore and is toxic to proliferating but not growth quiescent endothelial cells in vitro and inhibits angiogenesis in vivo [16]. When conjugated at the γ-glutamyl residue (with fluorophores or radioisotopes), the metabolism of GSAO by plasma membrane γ-glutamyltransferase (and subsequent transport into the cell and activation of the mitochondrial permeability transition pore) is prevented. In dying cells when the plasma membrane integrity has been compromised, GSAO conjugated at the γ-glutamyl residue enters intact and binds to intracellular proteins, predominantly 90-kDa heat shock proteins (hsp90). Labelling of apoptotic cells with conjugated GSAO is co-incident with Annexin V, propidium iodide and Sytox Blue [17]. Hsp90 is the most abundant member of the 90-kDa molecular chaperone family of proteins [18] and in malignancy there is greater than normal abundance of hsp90 [19] thus making it an attractive target for cell death imaging in oncology. In vivo, fluorophore-labelled GSAO localized to cells that subsequently stained with an antibody for activated caspase 3 but not to cells that did not stain for activated caspase 3. GSAO labelled with ^111^In labelled apoptotic cells but not viable cells in vitro and in vivo. [^111^In]In-DTPA-GSAO demonstrated uptake into dead and dying cells in subcutaneous tumours in a pattern concordant with [^99m^Tc]Tc-Annexin V. [^111^In]In-DTPA-GSAO was also seen in normal organs, especially the kidneys with lesser amounts in the liver and spleen [17]. In view of these observations, radiolabelled GSAO represents a potentially novel method for in vivo imaging of cell death. This study assessed the biodistribution and tumour uptake of GSAO labelled with ^111^In and ^67^Ga.

## Methods

### Preparation of DTPA GSAO and DOTA GSAO

Diethylene triamine pentaacetic acid GSAO (DTPA-GSAO) was synthesized as previously described [17]. Stocks of this were diluted to a concentration of 1 mg/mL and 46 μg aliquots stored at − 20 °C in nitrogen filled glass vials.

Preparation of 1,4,7,10-tetraazacyclododecane-1,4,7,10-tetraacetic acid GSAO (DOTA-GSAO) was undertaken by dissolving GSAO (155 mg, 0.28 mmol) in 20 mL of argon purged water. While stirring under argon, the pH of the solution was adjusted to 7.2 by slow addition of 0.1 M NaHCO_3_. DOTA-NHS (258 mg, 0.34 mmol) (Macrocyclics) was added in two equal portions with a pH adjustment to 7.0 after each addition using 0.1 M NaHCO_3_. The reaction mixture was stirred for 30 min at room temperature following the final addition. The crude product solution was purified on a Biotage RP18 Cartridge (YMC-GEL, ODS-AQ, 120-S50) with water-acetonitrile gradient as eluant to yield 99% pure product (105 mg, 40% yield) upon lyophilization.

### Radiolabelling of DTPA GSAO and DOTA GSAO

Labelling of DTPA-GSAO with ^111^In was performed as described previously [17].

DOTA-GSAO was labelled with ^67^Ga by preparing a 1-mM solution of DOTA GSAO. To 200 μL of this solution was added 4 mL of CH_3_COONH_4_ buffer (pH 4.8, 0.05 M). Seven hundred forty megabecquerels of [^67^Ga]GaCl_3_ in approximately 400 μL of 0.1 M HCl was added and the mixture incubated at 100 °C for 30 min. Quality control was performed by ITLC-SG (Varian) developed in 0.1 M trisodium citrate, pH 5.0 (2.94 g trisodium citrate dihydrate was dissolved in 95 mL distilled water, the pH adjusted to 5.0 with 6.0 M HCl and then distilled water added to a total volume of 100 mL). The ITLC-SG paper was cut into 10 1-cm segments and these were counted in a well counter (2480 Wizard 2, Perkin Elmer). Stability was assessed up to 24 h post radiolabelling by incubation in human plasma at 37 °C.

### Biodistribution of [^111^In]In-DTPA-GSAO and [^67^Ga]Ga-DOTA-GSAO

All studies were performed with prior approval of the University of New South Wales, Animal Care and Ethics Committee (11/35B, 11/69B, 11/103A) and conducted in full compliance with institutional and national guidelines. Biodistribution of [^111^In]In-DTPA-GSAO and [^67^Ga]Ga-DOTA-GSAO were performed in balb c nu/nu male mice bearing subcutaneous PC3M-luc-C6 xenografts. Prior to performance of these biodistribution studies, cell death of PC3M-luc-C6 cells (in particular the ability of conjugates of GSAO to detect cell death in this cell line) was characterized in vitro and in vivo (see Additional file 1). PC3M-luc-C6 cells (Caliper LifeSciences) were cultured in Roswell Park Memorial Institute medium (RPMI) medium supplemented with 10% foetal bovine serum, 2 mM L-glutamine and 1 μg per mL penicillin/streptomycin. Cell culture plasticware was from Techno Plastic Products (Trasadingen). All other cell culture reagents were from Gibco. PC3M-luc-C6 cells were implanted by subcutaneous injection of 3 × 10^6^ cells (100 μL) in the interscapular region of balb c nu/nu male mice. Tumours were allowed to grow for 21–28 days.

[^111^In]In-DTPA-GSAO or [^67^Ga]Ga-DOTA-GSAO was administered intravenously via the tail vein to each mouse. All post injection syringes were retained and measured in dose calibrator (CRC-25R, Capintec) to determine residual activity. Mice were placed into individual cages with an impervious absorbent liner (for collection of activity excreted in urine and faeces) immediately following tracer administration. Mice were divided into 5 subgroups and tracer biodistribution was allowed to occur for an average of 1.5 h (1.3–1.7), 2.7 h (2.5–3.0), 5.3 h (4.8–5.7), 8.0 h (7.9–8.1) and 24.1 h (23.9–24.5) in each cohort. There were four mice at each subgroup for each radiopharmaceutical, except for the subgroup at 5.3 h for [^111^In]In-DTPA-GSAO which contained 6 mice (i.e. in total 22 mice were included in the [^111^In]In-DTPA-GSAO group and 20 mice were included in the [^67^Ga]Ga-DOTA-GSAO group). The weight for the mice in the [^111^In]In-DTPA-GSAO group was 19.7 ± 1.6 g (mean ± SD) and for the [^67^Ga]Ga-DOTA-GSAO group was 18.6 ± 2.5 g (mean ± SD). Mice were provided with free access to food and water during the uptake period. Following the uptake period mice were sacrificed by lethal carbon dioxide overdose and blood samples immediately taken by cardiac puncture. Mice were then imaged on a γ camera (Discovery 670, GE Healthcare, GE Healthcare) using a pinhole collimator with a 2.5-mm pinhole insert 256 × 256 matrix for 300 s per view from the dorsal projection. A marker view was also performed using a cobalt point source to indicate the tip of the nose and base of the tail.

Immediately following imaging, mice were dissected and tissues and tumour harvested, weighed and counted in a γ counter (Wizard 2, Perkin Elmer), 60 s per sample, peaked for the appropriate radioisotope. The impervious padded liners used in each cage as well as all excreta were collected, sealed in a plastic bag and the activity measured in a dose calibrator.

Biodistribution was expressed as per cent of injected activity per gramme (%IA/g) ± standard deviation (SD) of tissue. For the calculation of administered activity, corrections were made for residual activity within the syringe following injection and all activities were decay corrected to the time of injection. All statistical analysis was performed using R (version 3.6.0, R Foundation for Statistical Computing). Comparisons of tumour and organ uptake were performed using Welch’s *t* test.

### Radiation dosimetry calculations

Estimated human radiation dosimetry was calculated for [^67^Ga]Ga-DOTA-GSAO. Animal data expressed as %IA/g was extrapolated to the human model using the kg/g method based on the organ weights for an ideal (73.7 kg) human male voiding model [20] using the following formula:

$$ {\left(\frac{\%}{\mathrm{organ}}\right)}_{\mathrm{human}}=\left[{\left(\frac{\%}{{\mathrm{g}}_{\mathrm{organ}}}\right)}_{\mathrm{animal}}\times {\left({\mathrm{kg}}_{\mathrm{TBweight}}\right)}_{\mathrm{animal}}\right]\times {\left(\frac{{\mathrm{g}}_{\mathrm{organ}}}{{\mathrm{kg}}_{\mathrm{TBweight}}}\right)}_{\mathrm{human}} $$

The %IA/g estimated for each organ was entered into OLINDA/EXM and biexponential curve fitting performed using the curve tools within OLINDA/EXM, and dose calculated by OLINDA/EXM [21]. Effective radiation dose for individual organs and for the whole body was expressed as mSv/MBq.

## Results

### Biodistribution and tumour uptake of [^111^In]In-DTPA GSAO and [^67^Ga]Ga-DOTA-GSAO

**Fig. 1:**
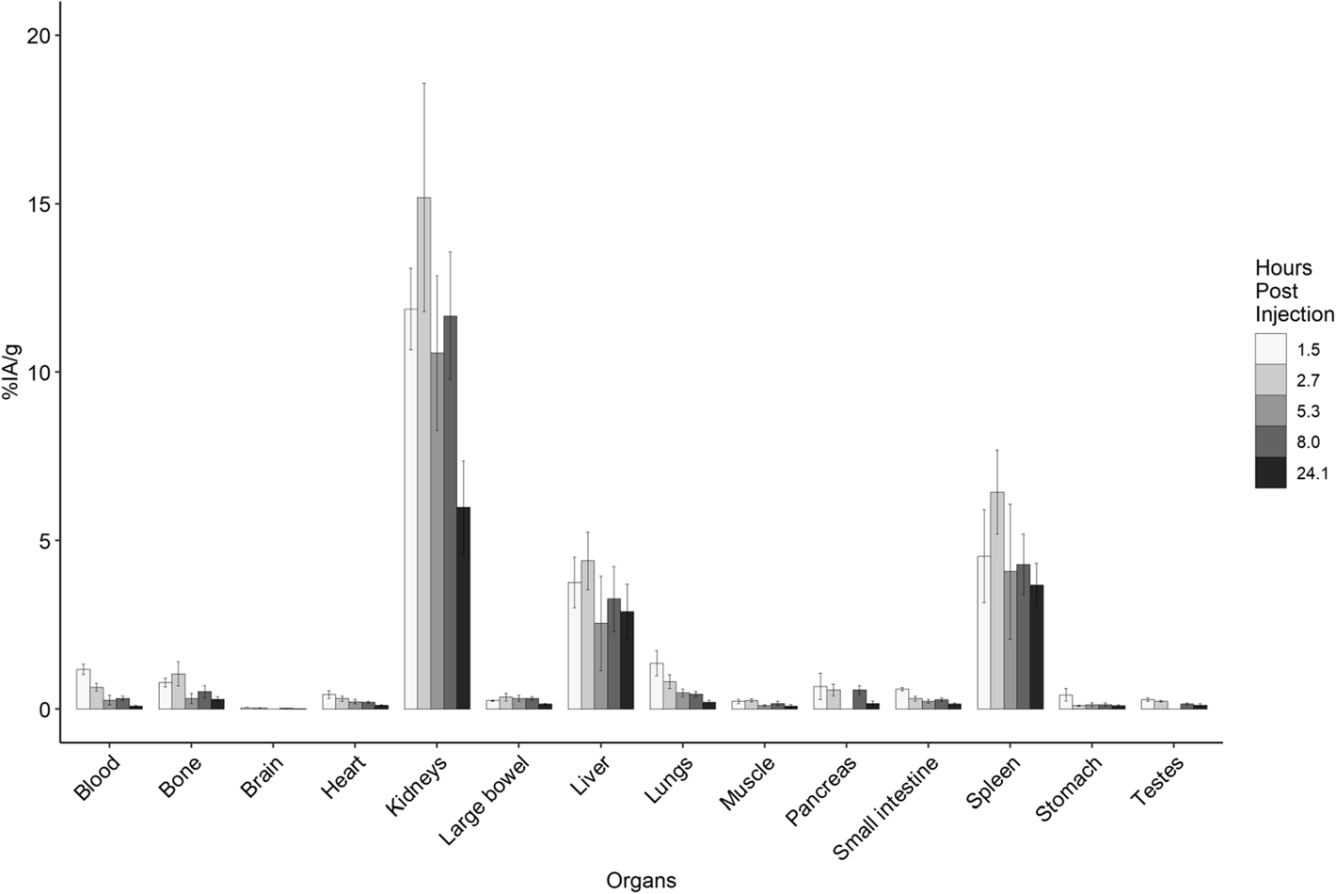
Biodistribution of [^111^In]In-DTPA-GSAO in mice. The data is from 4 biological replicates at each uptake time (except at 5.3 h which has 6 replicates) (mean ± SD)

Average radiochemical purity of [^111^In]In-DTPA-GSAO was 94% (92–95%). The average injected activity of [^111^In]In-DTPA-GSAO was 22.3 (range 18.5–27.9) MBq. The tissue biodistribution of [^111^In]In-DTPA-GSAO is shown in Fig. 1. Blood samples showed an early peak with progressive decline over later time points. In some organs especially the kidneys, liver and spleen there was maximum uptake at 2.7 h followed by decline at later time points. The organ with the highest concentration of [^111^In]In-DTPA-GSAO was in the kidneys (16.54 ± 3.86 %IA/g), followed by the spleen (6.44 ± 1.24 %IA/g) and liver (4.40 ± 0.86 %IA/g), all at 2.7 h post injection. There was low level tracer uptake into the small and large bowel and the spleen with minimal uptake into the other organs. There was relatively rapid urinary excretion of [^111^In]In-DTPA-GSAO with 21.91 ± 4.04 %IA retained at 2.7 h.

Uptake of [^111^In]In-DTPA-GSAO into dead and dying cells in tumour peaked at 2.44 ± 0.26 %IA/g at 2.7 h post injection with a slow decline at later time points. The tumour to blood ratio increased throughout the period of observation. At peak tumour uptake the tumour to blood ratio was 3.72 ± 1.09 (Fig. 3).

**Fig. 2:**
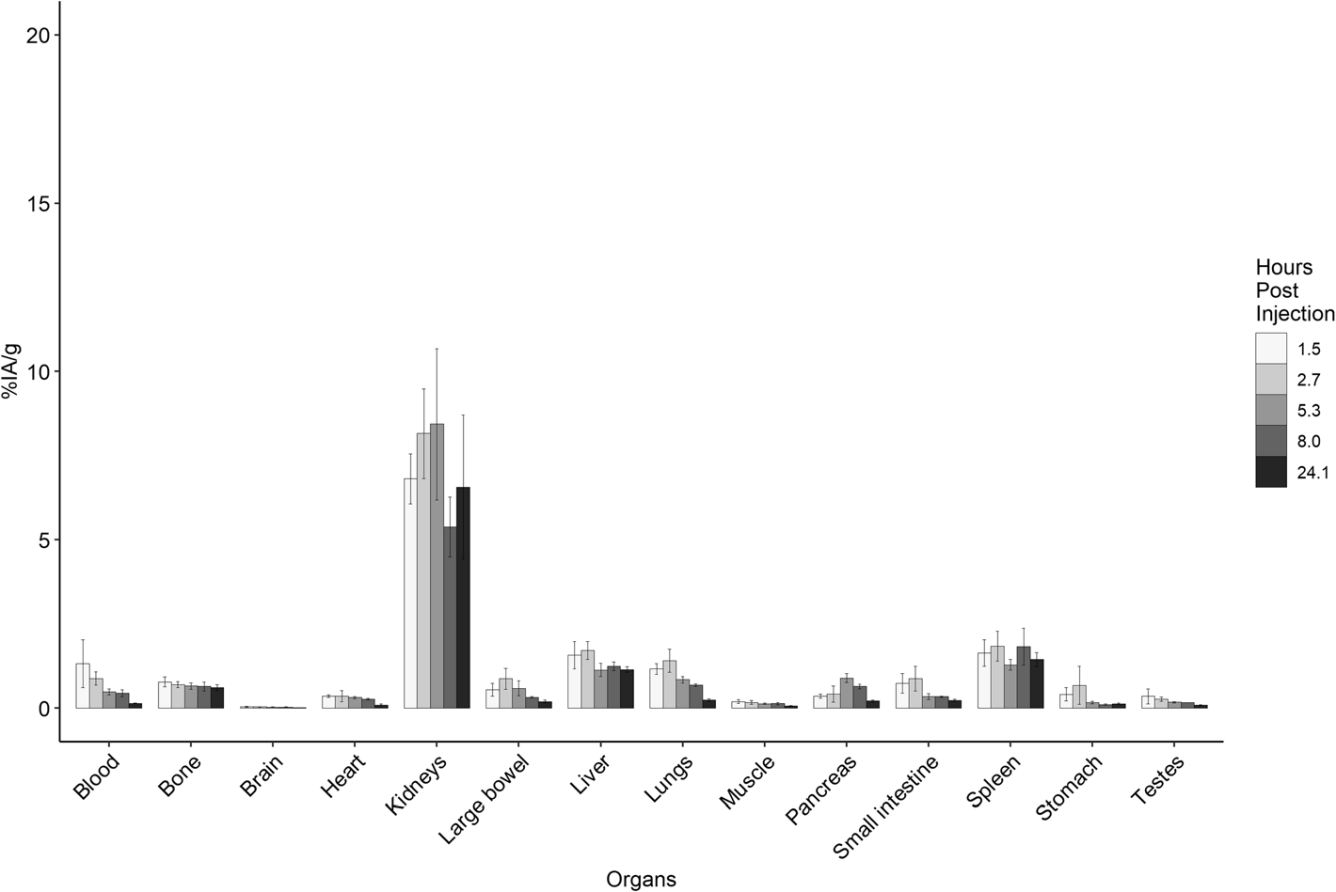
Biodistribution of [^67^Ga]Ga-DOTA-GSAO in mice. The data is from 4 biological replicates at each uptake time (mean ± SD)

Average radiochemical purity of [^67^Ga]Ga-DOTA-GSAO was 95% (92–98%). There was minimal decline in [^67^Ga]Ga-DOTA-GSAO radiochemical purity in human plasma up to 24 h following labelling which was 86.7 ± 0.65% (mean ± SD of two separate experiments). The mean injected activity of [^67^Ga]Ga-DOTA-GSAO was 18.8 (14.2–20.8) MBq. The organ biodistribution of [^67^Ga]Ga-DOTA-GSAO is shown in Fig. 2. Activity in blood shows an early peak with gradual decline over time. In contrast, most organs demonstrate peak uptake at 2.7 h (except for the liver where uptake is marginally higher at 5.3 h) followed by a slow decline over time. The organs with the highest concentration of [^67^Ga]Ga-DOTA-GSAO at 2.7 h were the kidneys (8.16 ± 1.33 %IA/g), followed by the spleen (1.85 ± 0.44 %IA/g) and the liver (1.72 ± 0.27 %IA/g). There were lower levels of uptake in the small and large bowel (0.87 ± 0.31 %IA/g). [^67^Ga]Ga-DOTA-GSAO was rapidly excreted in urine and 15.70 ± 3.85 %ID was retained at 2.7 h.

**Fig. 3:**
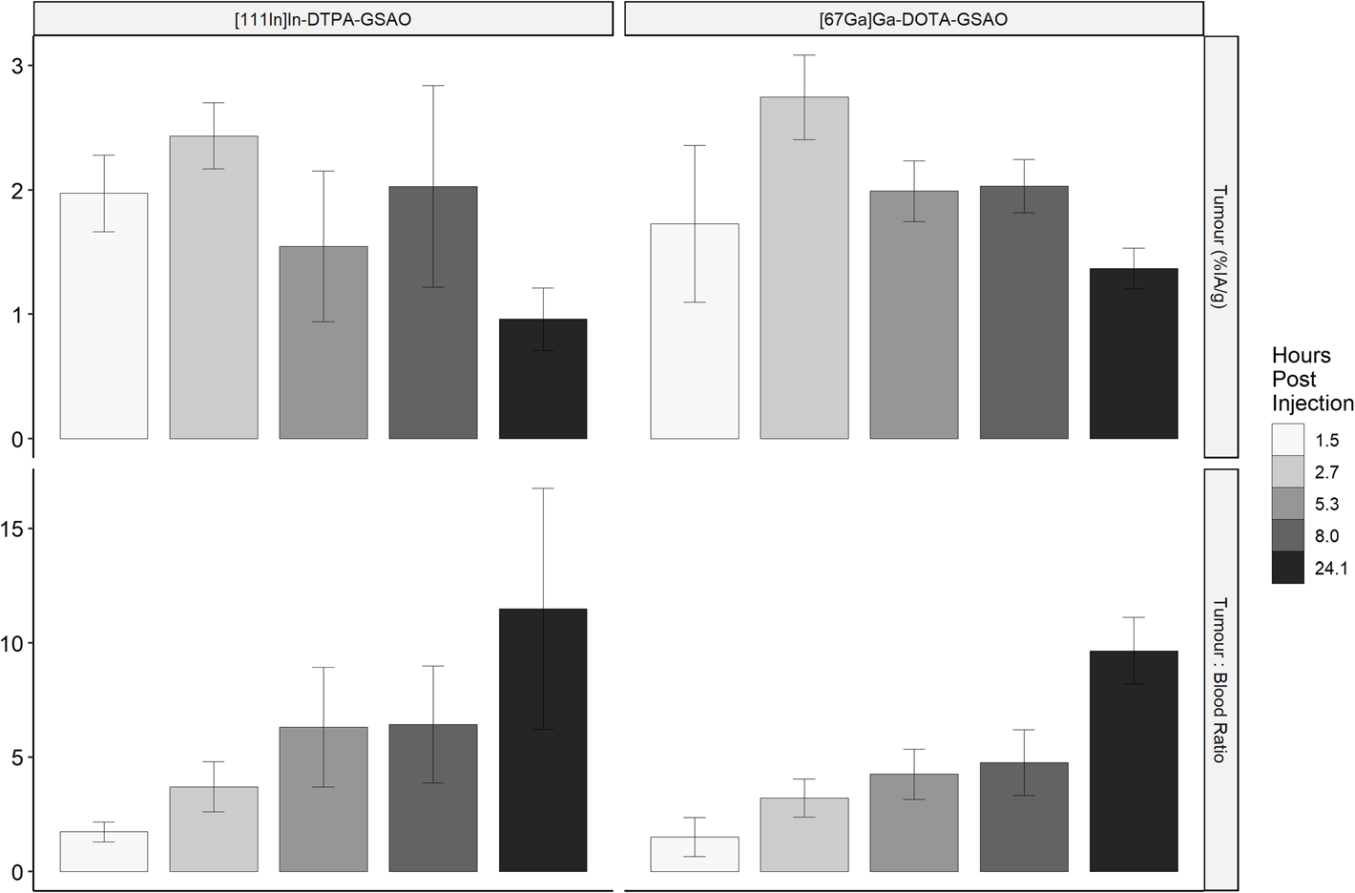
Tumour %IA/g and tumour: blood ratio for [^111^In]In-DTPA-GSAO and [^67^Ga]Ga-DOTA-GSAO in mice. The data is from 4 biological replicates at each uptake time (except at 5.3 h for [^111^In]In-DTPA-GSAO which has 6 replicates) (mean ± SD)

Uptake of [^67^Ga]Ga-DOTA-GSAO into dead and dying cells in tumour peaked at 2.75 ± 0.34 %IA/g at 2.7 h post injection with a slow decline at later time points. The tumour to blood ratio increased throughout the period of observation. At peak tumour uptake, the tumour to blood ratio was 3.22 ± 0.83 (Fig. 3).

### Comparison of [^111^In]In-DTPA-GSAO and [^67^Ga]Ga-DOTA-GSAO

**Fig. 4:**
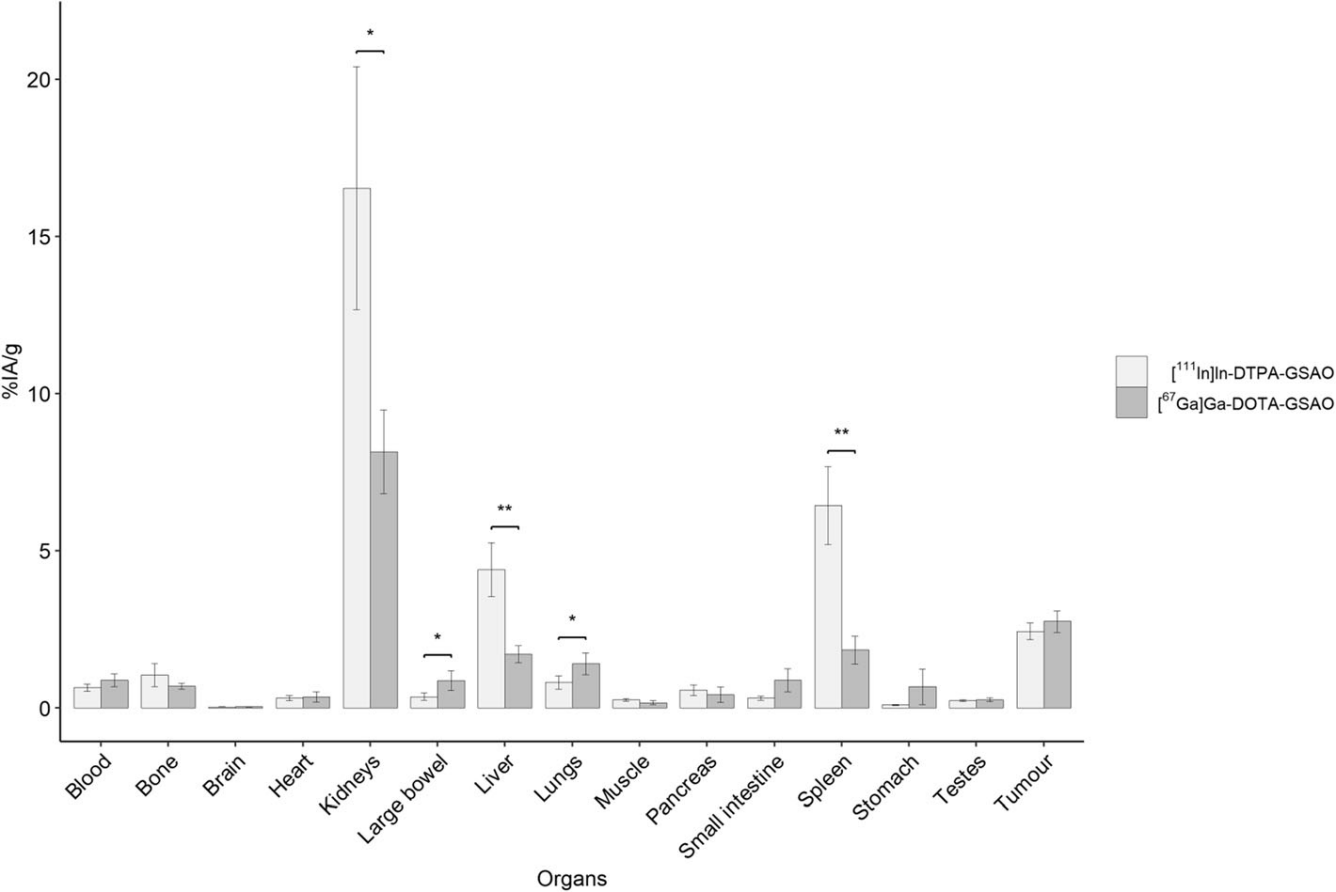
Comparison of biodistribution of [^111^In]In-DTPA-GSAO and [^67^Ga]Ga-DOTA-GSAO at 2.7 h post administration in mice (*p ≤ 0.05, **p ≤ 0.01). The data is from 4 biological replicates (mean ± SD)

**Fig. 5:**
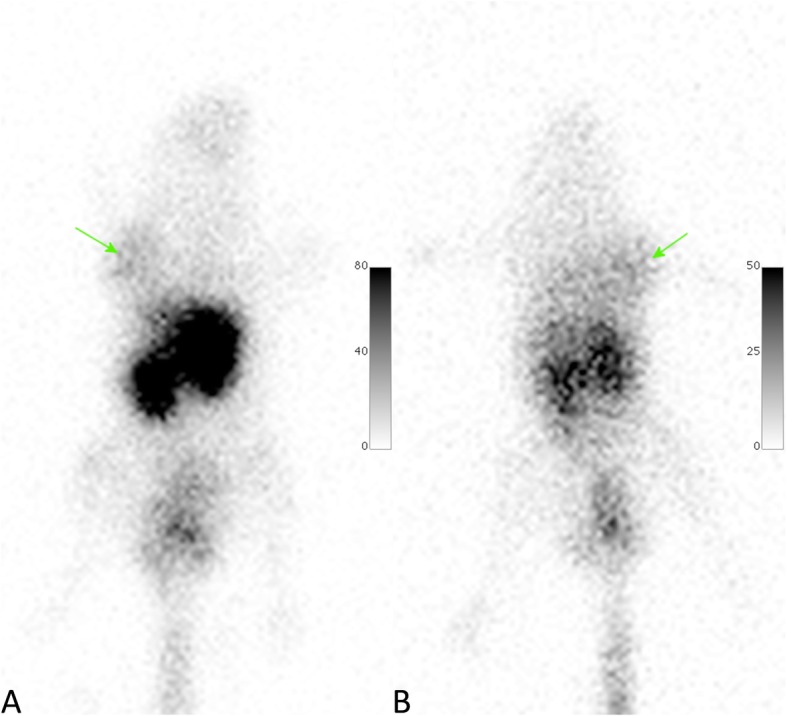
Dorsal pinhole images of [^111^In]In-DTPA-GSAO (**a**) and [^67^Ga]Ga-DOTA-GSAO (**b**) at 2.7 h post administration in mice. Arrow indicate the location of the subcutaneous tumour xenografts

Peak tumour uptake for both [^111^In]In-DTPA-GSAO and [^67^Ga]Ga-DOTA-GSAO occurred at 2.7 h post injection and there was no significant difference in mean tumour uptake between these two tracers (*p* = 0.23). However, at 2.7 h post injection there was significantly lower [^67^Ga]Ga-DOTA-GSAO uptake in the kidneys (*p* = 0.017) liver (*p* = 0.006) and spleen (*p* = 0.003). There was higher [^67^Ga]Ga-DOTA-GSAO uptake in the lungs (*p* = 0.031) and large bowel (*p* = 0.038) (Fig. 4). At 2.7 h there was greater clearance of [^67^Ga]Ga-DOTA-GSAO (15.70 ± 3.85 %IA retained) than [^111^In]In-DTPA-GSAO (21.91 ± 4.04 %IA retained); however, the difference was not significant (*p* = 0.068) (Fig. 5).

### Estimation of human dosimetry for [^67^Ga]Ga-DOTA-GSAO

The estimated human total body effective dose for [^67^Ga]Ga-DOTA-GSAO is 1.54E− 02 mSv/MBq, which would give a total body effective dose of 3.08–6.16 MBq assuming administration of 200–400 MBq of [^67^Ga]Ga-DOTA-GSAO. Estimated individual organ doses are listed in Table 1.

**Table 1:** Estimated individual organ dosimetry (mSv/MBq) for [^67^Ga]Ga-DOTA-GSAO

Target organ	Alpha	Beta	Photon	Total	ED
Adrenals	0.00E000	1.23E− 03	1.22E− 02	1.34E− 02	3.36E− 05
Brain	0.00E000	5.42E− 05	8.80E− 04	9.34E− 04	2.34E− 06
Breasts	0.00E000	1.23E− 03	1.97E− 03	3.20E− 03	1.60E− 04
Gallbladder wall	0.00E000	1.23E− 03	1.13E− 02	1.25E− 02	0.00E000
Lower large intestine wall	0.00E000	9.13E− 03	7.19E− 03	1.63E− 02	1.96E− 03
Small intestine	0.00E000	9.44E− 03	8.18E− 03	1.76E− 02	4.40E− 05
Stomach wall	0.00E000	2.56E− 03	6.84E− 03	9.40E− 03	1.13E− 03
Upper large intestine wall	0.00E000	1.23E− 03	8.09E− 03	9.32E− 03	2.33E− 05
Heart wall	0.00E000	4.42E− 04	4.27E− 03	4.71E− 03	0.00E000
Kidneys	0.00E000	1.58E− 01	5.43E− 02	2.12E− 01	5.31E− 03
Liver	0.00E000	1.17E− 02	1.35E− 02	2.52E− 02	1.26E− 03
Lungs	0.00E000	6.55E− 04	3.69E− 03	4.34E− 03	5.21E− 04
Muscle	0.00E000	1.23E− 03	3.89E− 03	5.12E− 03	1.28E− 05
Ovaries	0.00E000	1.23E− 03	6.82E− 03	8.06E− 03	0.00E000
Pancreas	0.00E000	6.38E− 03	1.15E− 02	1.79E− 02	4.48E− 05
Red marrow	0.00E000	1.07E− 03	4.91E− 03	5.98E− 03	7.18E− 04
Osteogenic cells	0.00E000	9.15E− 03	6.15E− 03	1.53E− 02	1.53E− 04
Skin	0.00E000	1.23E− 03	1.99E− 03	3.23E− 03	3.23E− 05
Spleen	0.00E000	1.31E− 02	1.39E− 02	2.70E− 02	6.75E− 05
Testes	0.00E000	5.14E− 03	3.71E− 03	8.85E− 03	1.77E− 03
Thymus	0.00E000	1.23E− 03	2.72E− 03	3.95E− 03	9.88E− 06
Thyroid	0.00E000	1.23E− 03	2.38E− 03	3.61E− 03	1.80E− 04
Urinary bladder wall	0.00E000	2.53E− 02	1.46E− 02	3.99E− 02	2.00E− 03
Uterus	0.00E000	1.23E− 03	7.78E− 03	9.01E− 03	2.25E− 05
Total body	0.00E000	2.35E− 03	4.43E− 03	6.77E− 03	0.00E000

*ED* effective dose

## Discussion

As cell death is such a ubiquitous process in health and disease, the ability to image cell death in vivo has significant potential clinical utility. In oncology particularly, the evasion of cell death by apoptosis is a hallmark of cancer and many therapies especially cytotoxic chemotherapy and radiotherapy act by induction of apoptosis. The ability to image cell death in vivo would potentially provide an earlier and more specific assessment of treatment response than currently available anatomic and molecular imaging techniques.

Fluorophore-labelled conjugates of GSAO have been shown to be able to image cyclophosphamide-induced tumour cell death in murine orthotopic human mammary tumours. There was a significant increase in the GSAO fluorescence signal in the treated tumours measured in vivo and ex vivo and the signal co-localized with apoptotic cells in sectioned tumours [22]. In another study, fluorophore-labelled conjugates of GSAO localized to murine cerebral cryolesions and the areas of fluorescence corresponded with areas of TUNEL staining ex vivo [23].

However, fluorescence imaging is not suitable for imaging of cell death in clinical practice, leading to the investigation of radiolabelled conjugates of GSAO. This study reports on two radiolabelled conjugates of GSAO, [^111^In]In-DTPA-GSAO and [^67^Ga]Ga-DOTA-GSAO (a subset of this data was presented previously [24]). Both agents demonstrated uptake into tumours comparable to that reported previously [17], with slightly greater uptake of [^67^Ga]Ga-DOTA-GSAO into tumour, peaking at 2.7 h post injection, a convenient time point for imaging. The tumour to blood ratio increased progressively, consistent with specific, high affinity binding to dead and dying cells in tumour and clearance of non-specific activity within blood. Importantly, compared with [^111^In]In-DTPA-GSAO, [^67^Ga]Ga-DOTA-GSAO uptake into dead and dying cells in tumour was higher than all normal organs except for the kidneys. Comparison of uptake of GSAO with published data for other radiopharmaceuticals for the imaging of dead and dying cells is difficult due to the heterogeneity of the models and methods used. However, in two studies, direct comparison was undertaken between [^99m^Tc]Tc-Annexin V and [^111^In]In-DTPA-GSAO. Park and colleagues demonstrated that [^111^In]In-DTPA-GSAO and [^99m^Tc]Tc-Annexin V had qualitatively similar uptake within tumour using dual energy SPECT CT [17]. Tahara and colleagues performed in vivo and ex vivo SPECT CT in a rabbit and mouse myocardial infarction model and demonstrated uptake of [^111^In]In-DTPA-GSAO in the same areas of myocardial infarction as [^99m^Tc]Tc-Annexin A5 and found [^111^In]In-DTPA-GSAO uptake to be more intense than [^99m^Tc]Tc-Annexin A5 [25].

In terms of normal tissue biodistribution, both [^111^In]In-DTPA-GSAO and [^67^Ga]Ga-DOTA-GSAO are renally excreted with the highest concentration of uptake in the kidneys. [^67^Ga]Ga-DOTA-GSAO was more rapidly excreted than [^111^In]In-DTPA-GSAO, although the difference was not statistically significant. The liver and spleen were the organs with the next greatest uptake of both [^111^In]In-DTPA-GSAO and [^67^Ga]Ga-DOTA-GSAO, although hepatic and splenic uptake was significantly less for [^67^Ga]Ga-DOTA-GSAO than [^111^In]In-DTPA-GSAO. Thus overall, both agents appear to be suitable for further studies of imaging of cell death in vivo, although the slightly higher tumour uptake and lower normal tissue uptake of [^67^Ga]Ga-DOTA-GSAO may make it the preferred agent.

Several different radiopharmaceuticals have been developed for imaging cell death, the most extensively studied being ^99m^Tc-labelled Annexin V. Small human studies have demonstrated a significant correlation between tumour uptake following commencement of treatment and response to therapy [5, 7]. However, [^99m^Tc] Tc Annexin V has high uptake in the kidneys (49.7 %IA/g) and liver (13.1 %IA/g), which may impair its ability to image cell death in these organs [9]. Uptake in these organs was much greater than for either of the GSAO conjugates in this study. Annexin V has also been labelled with other single photon and positron-emitting isotopes. Qin and colleagues examined Annexin V, labelled with ^18^F ([^18^F]F-rh-His_10_-Annexin V) and observed rapid renal excretion and much lower levels of uptake in normal organs, similar to that demonstrated with the GSAO conjugates in this study. However, synthesis and purification of [^18^F]F-rh-His_10_-Annexin V is complex [26]. Other methods have also been investigated to target phosphatidyl serine including synaptotagmin I and phosphatidylserine binding peptides. A recent review concluded that [^99m^Tc]Tc-annexin V remains the radiotracer of choice for imaging cell death by targeting phosphatidylserine, but Annexin V-based radiotracers have a number of limitations including complex and expensive radiolabelling, poor patterns of biodistribution, slow blood clearance and poor tissue penetration [10].

Another approach to imaging cell death has been with radiolabelled caspase 3/7 inhibitors. Human biodistribution of [^18^F]ICMT-11, a caspase 3-specific PET tracer has been studied and demonstrated high levels of uptake in the liver, biliary system and small bowel. It was observed that the main routes of excretion were through the renal and hepatobiliary system; however, only 18% of activity was renally excreted in the first 4 h with high levels of uptake in the hepatobiliary system and slow washout through the gastro-intestinal tract [12]. The clearance of [^18^F]ICMT-11 was much slower and the hepatobiliary and gastro-intestinal localisation much greater than observed with the GSAO conjugates examined in this study. In addition, no increase in [^18^F]ICMT-11 tumour uptake was observed in patients with lung or breast cancer following commencement of chemotherapy [13].

A further approach to imaging cell death has been with [^18^F]2-(5-fluoropentyl)-2-methyl malonic acid ([^18^F]ML10), which accumulates within apoptotic cells driven by apoptotic scramblase activation, depolarization and cellular acidification [27]. [^18^F]ML10 has been shown to have fast clearance from the blood and other organs (elimination half time of 1.3 and 1.1 h, respectively) through urinary excretion [28]. Some studies have demonstrated the ability of [^18^F]ML10 to detect cell death in response to treatment [29, 30], however in a recent study using a rat stroke model, fluorinated caspase 3/7 inhibitor but not [^18^F]ML10 demonstrated uptake in areas of ischaemic brain compared with the contralateral side, calling into question the ability of [^18^F]ML10 to specifically detect dead and dying cells [31].

In comparison with other radiopharmaceuticals investigated for imaging cell death, the biodistribution of the GSAO conjugates investigated herein appears to be relatively favourable. While both [^111^In]In-DTPA-GSAO and [^67^Ga]Ga-DOTA-GSAO demonstrate high levels of renal uptake and are excreted in urine, which may potentially interfere with image interpretation in the abdomen and pelvis, renal uptake is less than that seen with [^99m^Tc] Tc Annexin V. Compared to [^18^F]ICMT-11, there is much less hepatic uptake and no gastro-intestinal excretion, making GSAO-based radiopharmaceuticals much more suitable for imaging in the abdomen and pelvis than [^18^F]ICMT-11. In addition, renal uptake and urinary excretion is commonly seen with a wide range of single photon- and positron-emitting radiopharmaceuticals which are in routine clinical use but this has not impaired there clinical utility in the majority of applications.

The half-life of ^67^Ga and ^111^In used in this study is too long to be useful for rapid assessment of changes in cell death in response to treatment, and isotopes with shorter half-lives would be more appropriate for clinical translation. While short half-life single photon-emitting isotopes such as ^99m^Tc are widely available and would be appropriate, attempts to conjugate ^99m^Tc to GSAO have been unsuccessful. Additionally, positron-emitting isotopes would be preferable for more accurate quantitative imaging. Investigations have been undertaken to conjugate ^68^Ga to GSAO. While DOTA is an appropriate bifunctional chelator for ^68^Ga, since commencing this work other bifunctional chelators have become available which are superior with higher affinity and offer rapid room temperature labelling. Recently, we reported a method for rapid, room temperature conjugation of GSAO with ^68^Ga using 2,2′-(7-(1-carboxy-4-((2,5-dioxopyrrolidin-1-yl)oxy)-4-oxobutyl)-1,4,7-triazonane-1,4-diyl) diacetic acid (NODAGA) suitable for clinical translation [32]. A first in human study of [^68^Ga]Ga-NODAGA-GSAO (now referred to as Cell Death Indicator [CDI) PET is now underway.

## Conclusions

Cell death imaging has the potential to be a clinically useful technique in a wide range of diseases, particularly for the assessment of treatment response in oncology. Although several agents have been investigated, no ideal agent has yet been identified due to sub-optimal biodistribution profiles and complex synthesis and radiolabelling. The GSAO conjugates used in this study have been previously shown to reliably detect dead and dying cells in vivo, and this study demonstrated favourable biodistribution characteristics, especially for [^67^Ga]Ga-DOTA-GSAO. However, the long half-life (78 h) of ^67^Ga is not suitable for translation into clinical practice and investigation of ^68^Ga-labelled GSAO is underway.

## Supplementary information

**Additional file 1.** Characterisation of cell death detected by GSAO in PC3M-luc-C6 cells and xenografts.

## Data Availability

All data generated or analysed during this study are included in this published article.

## Abbreviations

%IA: Per cent of injected activity
%IA/g: Per cent of injected activity per gramme
DOTA: 1,4,7,10-Tetraazacyclododecane-1,4,7,10-tetraacetic acid
DTPA: Diethylene triamine pentaacetic acid
GSAO: 4-(N-(S-glutathionylacetyl)amino) phenylarsonous acid
hsp90: 90-kDa heat shock proteins
SD: Standard deviation
