# Supplementary material for: Biodistribution and imaging of an hsp90 ligand labelled with 111In and 67Ga for imaging of cell death
Source: EJNMMI Res. 2020 Jan 20;10:4. doi: 10.1186/s13550-020-0590-x (PMC6971215; doi:10.1186/s13550-020-0590-x)
Supplement: Supplementary file 1 — Additional file 1. Characterisation of cell death detected by GSAO in PC3M-luc-C6 cells and xenografts. [file 13550_2020_590_MOESM1_ESM.docx]

# Additional file to “Biodistribution and imaging of an hsp90 ligand labelled with ^111^In and ^67^Ga for imaging of cell death”: Characterisation of cell death detected by GSAO in PC3M-luc-C6 cells and xenografts.

Ivan Ho Shon^1,2,3^

Divesh Kumar^4^

Chithradevi Sathiakumar^5^

Paula Berghofer^6^

Khang Van^5^

Andrew Chicco^7^

Philip J Hogg^2^

^1^Department of Nuclear Medicine and PET, Prince of Wales Hospital, NSW 2031, Australia

^2^The Centenary Institute, NHMRC Clinical Trials Centre, Sydney Medical School, University of Sydney NSW 2006, Australia

^3^Prince of Wales Clinical School, University of New South Wales, NSW, 2052 Australia

^4^Department of Nuclear Medicine and PET, Fiona Stanley Hospital, Murdoch, WA, 6150 Australia

^5^Department of Nuclear Medicine and PET, Liverpool Hospital, NSW 2170, Australia

^6^LifeSciences Division, Australian Nuclear Science and Technology Organisation, New Illawarra Road, Lucas Heights NSW 2234 Australia

^7^Department of Medical Physics, Westmead Hospital, NSW 2145, Australia

## In vitro assessment of PC3M-luc-C6 cell death

PC3M-luc-C6, a luciferase-expressing cell line of metastatic human prostate adenocarcinoma[1] was selected to allow for potential future correlations between bioluminescence and apoptotic signals. In vitro assessments of cell death of this cell line were performed to confirm that they are similar to the cell lines used in previous studies. PC3M-luc-C6 cells (Caliper LifeSciences) were cultured in Roswell Park Memorial Institute medium (RPMI) medium supplemented with 10% foetal bovine serum, 2 mM L-glutamine, and 1 µg per mL penicillin / streptomycin. Cell culture plasticware was from Techno Plastic Products (Trasadingen). All other cell culture reagents were from Gibco.

PC3M-luc-C6 were seeded at a density of 1 x 10^6^ cells and incubated with staurosporine (Sigma-Aldrich) at a final concentration of 4 µM or control (RPMI) for 4, 8 and 24 hours. Cells were then detached with trypsin, washed twice in ice-cold phosphate-buffered saline (PBS) then resuspended in ice-cold 0.1 M Hepes, pH 7.4 buffer containing 0.14 M NaCl and 2.5 mM CaCl_2_. An aliquot was taken and serially diluted in duplicate (2 x 10^5^ cells / well to 2.5 x 10^4^ cells / well) in a 96 well plate. 100 µl of luciferin (Gold Biotechnology) at a concentration of 300 µg/mL was then added. The cells were incubated for 10 min before being read in a luminometer (Promega). Bioluminescence was expressed as a percentage of baseline bioluminescence of untreated cells at 0 hours.

The remaining cells then underwent flow cytometry analysis. GSAO linked to Cy5.5 prepared as described previously[2] was added and incubated for 15 min with shaking at room temperature. Cells were washed with ice-cold 0.1 M Hepes, pH 7.4 buffer containing 0.14 M NaCl and 2.5 mM CaCl_2_ and then incubated with 5 μL per 100 μL of Annexin V-APC (BD Pharmingen) and 1 μg per mL propidium iodide (Molecular Probes, Invitrogen) for 15 min in the dark. Flow cytometry was performed using a BD™ FACS Canto II Flow Cytometer (BD Biosciences) and data analysed using FlowJo software version 8.7.

## Preparation of the animal model

All studies were performed with prior approval of the University of New South Wales, Animal Care and Ethics Committee (11/35B, 11/69B, 11/103A) and conducted in full compliance with institutional and national guidelines. PC3M-luc-C6 cells were cultured as above and implanted by subcutaneous injection of 3 x 10^6^ cells (100 µL) in the interscapular region of balb c nu/nu male mice. Tumours were allowed to grow for 21-28 days.

## [^111^In]In-DTPA-GSAO and [^111^In]In-DTPA-GSCA uptake into PC3M-luc-C6 xenografts

In order to confirm that *in vivo* uptake of ^[111^In]In-DTPA-GSAO but not the control radiopharmaceutical ^[111^In]In-DTPA-GSCA into dying PC3M-luc-C6 tumour cells is similar to that previously observed with other cell lines, tumour uptake to studies were performed at 5 h post intravenous administration of ^[111^In]In-DTPA-GSAO or ^[111^In]In-DTPA-GSCA (6 mice each). All post injection syringes were retained and measured in dose calibrator (CRC-25R, Capintec) to determine residual activity. Mice were placed into individual cages with an impervious absorbent liner (for collection of activity excreted in urine and faeces) immediately following tracer administration. Following the uptake period, mice were sacrificed by lethal carbon dioxide overdose and blood samples immediately taken by cardiac puncture. Mice were then imaged on a γ camera (Discovery 670, GE Healthcare, GE Healthcare) using a pinhole collimator with a 2.5 mm pinhole insert 256 x256 matrix for 300 second per view from the dorsal projection. A marker view was also performed using a cobalt point source to indicate the tip of the nose and base of the tail.

Immediately following imaging, mice were dissected, tissues and tumour harvested, weighed and counted in a γ counter (Wizard 2, Perkin Elmer), 60 seconds per sample. The impervious padded liners used in each cage as well as all excreta were collected, sealed in a plastic bag and the activity measured in a dose calibrator.

## Results

### In vitro assessment of PC-3M-luc-C6 cell death

In vitro PC3M-luc-C6 cells demonstrated similar amounts and rates of cell death (Figure 1A) to that seen with Jurkat A3 (T cell leukaemia) and HT1080 fibrosarcoma studied previously. In addition, with increasing staurosporine induced cell death there is a commensurate decline in bioluminescence (Figure 1B) which may represent a useful correlate of cell viability and death for further investigation.

### In vivo assessment of PC-3M-luc-C6 cell death

In vivo, PC3M-luc-C6 tumour xenografts demonstrated uptake of [^111^In]In-DTPA GSAO but not [^111^In]In-DTPA GSCA (Figure 1C). The mean tumour uptake of [^111^In]In-DTPA GSAO was 1.53 ± 0.63 %IA/g (mean ± SD), whereas the mean tumour uptake of [^111^In]In-DTPA GSCA was 0.06 ± 0.03 %IA/g. The tumour to blood ratio of [^111^In]In-DTPA GSAO was 6.77 ± 3.32 whereas the mean tumour to blood ratio of [^111^In]In-DTPA GSCA was 0.48 ± 0.29.


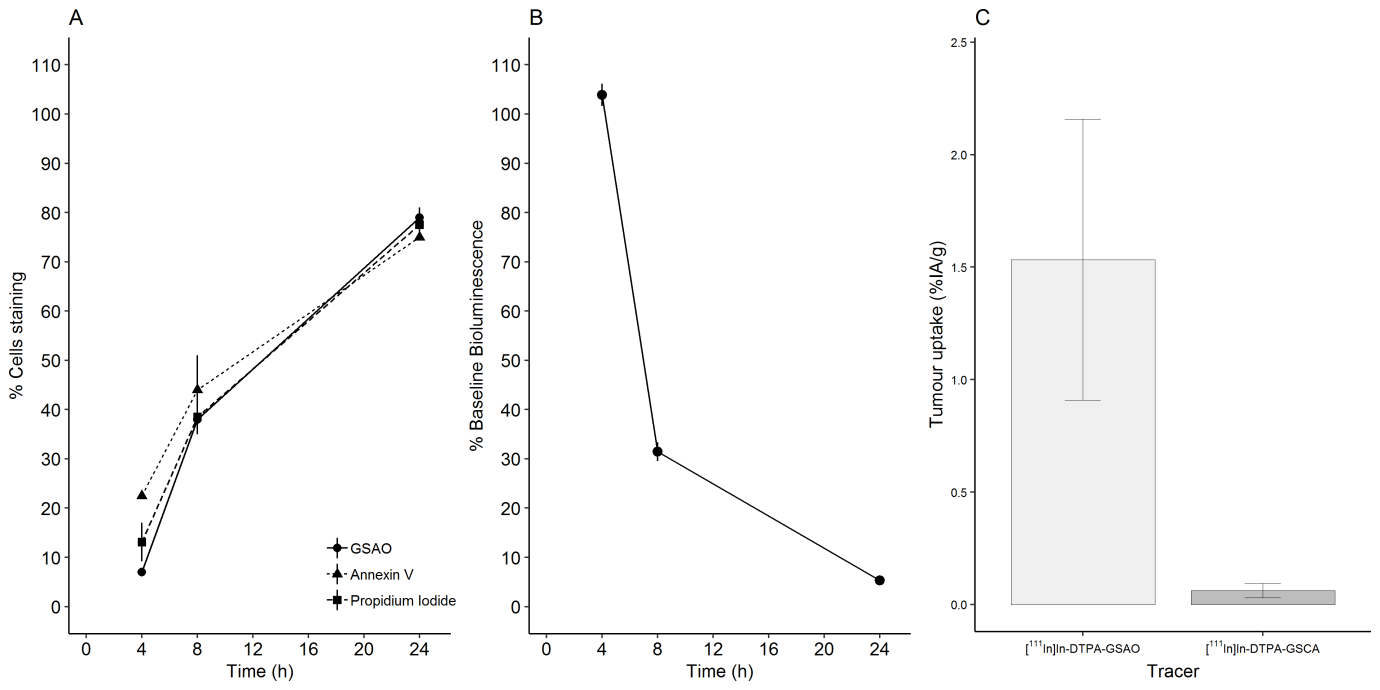


Figure 1: Cy5.5 GSAO shows concordant labelling of dead and dying PC3M-luc-C6 cells with Annexin V and Propidium iodide assessed by flow cytometry following treatment with 4 µM staurosporine for varying intervals up to 24 hours. Each data point represents the mean ± range of two separate experiments. (A). There is a commensurate decline in bioluminescence (expressed as a % of bioluminescence untreated controls) following treatment with 4 µM staurosporine for varying intervals up to 24 hours. Each data point represents the mean ± range of two separate experiments (B). In vivo uptake (expressed as mean %IA/g ± SD) of [^111^In]In-DTPA-GSAO and [^111^In]In-DTPA-GSCA into PC3M-luc-C6 tumour xenografts in balb c nu/nu mice at 5 hours post injection. Each bar represents the mean %IA/g ± SD of six separate measurements (C).

## References.

1. Jenkins DE, Oei Y, Hornig YS, Yu SF, Dusich J, Purchio T, et al. Bioluminescent imaging (BLI) to improve and refine traditional murine models of tumor growth and metastasis. Clin Exp Metastasis. 2003;20:733-44.

2. Park D, Don AS, Massamiri T, Karwa A, Warner B, MacDonald J, et al. Noninvasive imaging of cell death using an Hsp90 ligand. J Am Chem Soc. 2011;133:2832-5. doi:10.1021/ja110226y.
